# Tree-Based and Machine Learning Algorithm Analysis for Breast Cancer Classification

**DOI:** 10.1155/2022/6715406

**Published:** 2022-07-07

**Authors:** Arpit Bhardwaj, Harshit Bhardwaj, Aditi Sakalle, Ziya Uddin, Maneesha Sakalle, Wubshet Ibrahim

**Affiliations:** ^1^Department of Computer Science and Engineering, BML Munjal University, Kapriwas, Gurugram, Haryana, India; ^2^Department of Computer Science and Engineering, Galgotias University, Greater Noida, India; ^3^Department of Computer Science and Engineering, University School of Information and Communication Technology, Gautam Buddha University, Greater Noida, India; ^4^Department of Applied Sciences, SoEt, BML Munjal University, Kapriwas, Gurugram, Haryana, India; ^5^Department of Mathematics, Govt. S. N. P. G. College, Khandwa, India; ^6^Department of Mathematics, Ambo University, Ambo, Ethiopia

## Abstract

Breast cancer (BC) is the second leading cause of death in developed and developing nations, accounting for 8% of deaths after lung cancer. Gene mutation, constant pain, size fluctuations, colour (roughness), and breast skin texture are all characteristics of BC. The University of Wisconsin Hospital donated the WDBC dataset, which was created via fine-needle aspiration (biopsies) of the breast. We have implemented multilayer perceptron (MLP), K-nearest neighbor (KNN), genetic programming (GP), and random forest (RF) on the WBCD dataset to classify the benign and malignant patients. The results show that RF has a classification accuracy of 96.24%, which outperforms all the other classifiers.

## 1. Introduction

Millions of women worldwide are affected by breast cancer. Family history, hormones, and reproductive factors are all factors that can lead to breast cancer. Every year, one million women are diagnosed for the first time with breast cancer. Unfortunately, according to a study, half of them would die since doctors would not be able to diagnose cancer until it was too late. Despite the lack of data about the causes and treatments of breast cancer, the hypothesis states that any cancer originates due to uncontrolled cell development [1]. Any normal cell goes through a life cycle in which it divides to form new cells and then dies when the time comes. Any disruption in this life cycle raises cancer risk, and breast cancer is no exception. In addition, breast cancer strikes women more frequently as they age, regardless of their family history.

Researchers are concentrating their efforts on the early detection of breast cancer. It has the potential to boost diagnosis, treatment, and survival rates. Early detection is the most effective strategy to lessen the disease's health and economic implications, given the high cost of medication and the disease's importance. Because self-testing is infrequent, cancer is often discovered at an advanced stage.

Automated tools help experts detect specific diseases and make early diagnosis more feasible. The concept behind these systems is to analyse data in parallel in architecture that resemble the biological nervous system. ANNs can handle various tasks, including classification, defect detection, voice analysis, and incorrect input processing [2]. Innovative disease classification and detection strategies have been employed in several healthcare sectors. Artificial Neural Networks (ANNs) are a “hot” study subject in medicine because of their increased diagnostic accuracy, lower prices, and reduced human resources. ANNs are complex systems that are based on biological neuron networks. These networks estimate functions based on machine learning and cognitive sciences [3–5].

## 2. Related Work

Canedo and Marono [6] proposed the most refined 130, 99, and 102 attributes chosen using feature selection algorithms. The best result was 79% with the C4.5 decision tree algorithm utilizing the INT attribute selection approach. When the relief method uses feature removal methods, the Naive Bayes algorithm produces the best results (89%), and the SVM-RFE feature selection method produces the best results (90%). Compared to the approaches for picking characteristics, the k-S test technique is integrated with CFS. Then, in the test k-S - CFS, the selected methods are compared between CFS, MMR, Relief, and k-S, which are 80.5%, 87.4%, 82.4%, and 78.8%, respectively.

Amrane et al. [7] explore the Naïve Bayes (NB) and KNN classifier and use a cross-validation scheme for accuracy evaluation. In Naïve Bayesian Classifier (NBC), the variables are conditionally independent. Hence, Bayesian classifiers are best for compound datasets. On the other hand, in KNN, we use the Euclidian distance for evaluating the distances with other points. After comparing the results of both algorithms accurately, it has been found that the KNN algorithm has a greater accuracy of 95.71% than the NB algorithm, which has an accuracy of 96.19%. Still, if a large dataset is taken, then the running time taken for the KNN algorithm will increase in comparison with the NB algorithm.

Djebbari et al. [8] explore forecasting the survival time of breast cancer using machine learning. Their methodology exhibits improved precision compared to earlier outcomes using their breast cancer data.

Liu et al. [9] used decision trees based on unbalanced data to develop predictive models for 5-year survival rates of breast cancer. After preprocessing data from SEER breast cancer datasets, it is clear that the data distribution category is unbalanced. The prediction efficacies of combining the undersampling approach and decision tree are shown to balance the data after data preparation. The AUC of the model is 0.7484, with a 15% undersampling ratio. Model performance is the highest when the data distribution is about equal. The AUC is enhanced to 0.7678 after employing the bagging procedure.

Delen et al. [10] preclassified 202,932 breast cancer patient records in two segments: “survived” ones (93,273) and “did not survive” ones (109,659). The accuracy of the prediction of survivorship was in the region of 93%.

Aruna et al. [11] compared C4.5, NB, SVM, and KNN classifiers in WBC to find the best classifier. The WEKA tool was used for experiment conduction. The SVM is the most accurate classifier, with an accuracy of 96.99%.

Baboo and Sasikala [12] conducted a data mining survey using methodologies for gene selection categorization. This article focused on four essential emerging subjects, including the most commonly used machine learning approaches for gene selection and cancer categorization.

Here, we propose the classification of breast cancer using a machine learning algorithm, considering that these machine learning algorithms perform well in most pattern classification tasks.

## 3. Methodology

This section explains the dataset and the classification methods adopted.

### 3.1. Dataset

The tests were conducted with the WDBC dataset from the UCI repository. Authors frequently utilise this dataset based on human breast tissues to diagnose breast cancer diseases. The collection contains records of 32 tumour features derived from a digital image of a breast FNA in 569 patients. Cancer is represented by 30 of the 32 features. The topic ID and class label are represented by the remaining two. The classification label helps determine whether the subject is a benign or malignant tumour. In addition, ten cell nuclei attributes were acquired for each individual.

### 3.2. Classification Algorithms

The literature has a variety of breast cancer classification methods. This study classified the WDBC dataset using the MLP, KNN, GP, and RF algorithms. The classifiers are described further down.

#### 3.2.1. Multi-Layer Perceptron

A feed-forward artificial neural network called a multilayer perceptron (MLP) generates a set of outputs from a collection of inputs. An MLP is defined by numerous layers of input nodes coupled as a directed graph between the input and output layers. The MLP uses back propagation to train the network. The MLP is a method of deep learning. [13, 14].

#### 3.2.2. K-Nearest Neighbor

The KNN is a commonly used ML technique. It is a type of learning that occurs in conditions that do not require a learning phase. The model is created using the training sample, a distance function, and a class choice function based on the classes of the nearest neighbours. First, we must compare a new element to other elements using a similarity measure before classifying it. The element to be classified is then compared to its k-nearest neighbours, and the class with the most notable appearances among them is allocated to it. Finally, the neighbours are weighted based on the distance between them and the new items to categorise [15, 16].

#### 3.2.3. Genetic Programming

This paper offers GP as an evolutionary algorithm and an extension of GA as a foundation for feature generation. GA provides GP with the ability to choose features, but it is considerably broader. GP is beneficial for evaluating the efficiency of features and determining whether characteristics can survive the evolutionary process [17–21].

#### 3.2.4. Random Forest

Ho introduced random forest in 1995 to separate nodes for the first time. It is a collection of many decision trees that uses bootstrapping and random feature selection. Because it works well on massive datasets, random forest is a good fit for our investigation. Furthermore, a random forest is a classifier that uses a classification tree as its input, a vector of independently and identically distributed tree votes. As a result, the accuracy of a decision tree is more consistent and precise [22].

## 4. Experimental Results

**Figure 1:**
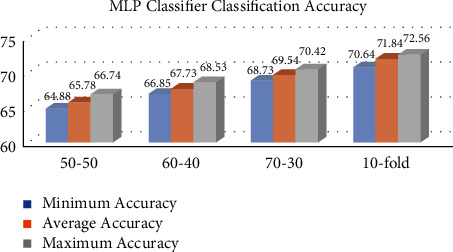
MLP classifier classification accuracy in %.

**Figure 2:**
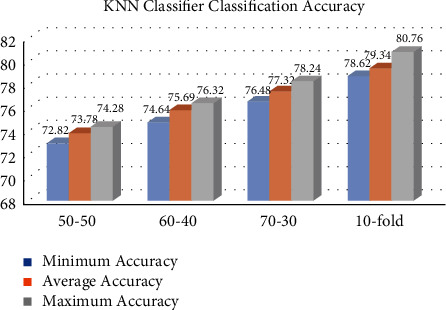
KNN classifier classification accuracy in %.

**Figure 3:**
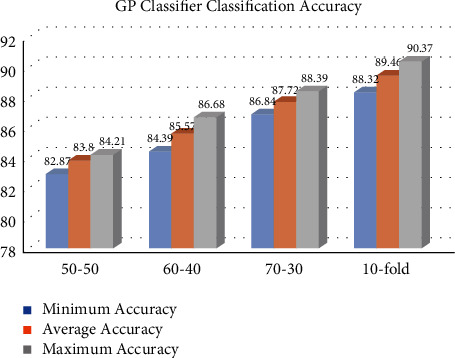
GP classifier classification accuracy in %.

**Figure 4:**
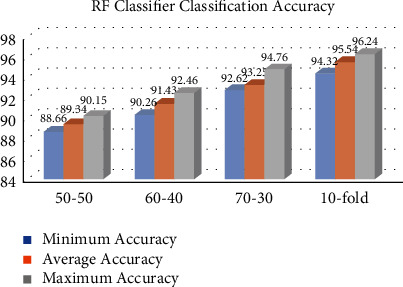
RF classifier classification accuracy in %.

This section describes the accuracy findings of the MLP, KNN, GP, and RF classifiers for breast cancer classification. Our dataset is partitioned into many partition schemes, with 569 samples. Figures 1–4 represent the MLP, KNN, GP, and RF classifier classification accuracy, respectively. The results show that the RF classifier outperforms the MLP, KNN, and GP classifiers. For example, for a 10-fold partition, the RF classifier's minimum, average, and maximum classification accuracy are 94.32%, 95.54%, and 96.24%, respectively. The performance measures of our implemented classifiers such as sensitivity, precision, specificity, and *F*1-score are compared in Table 1 on 50–50, 60–40, 70–30, and 10-fold partition schemes. The sensitivity, precision, specificity, and *F*1-score of the MLP classifier 10-fold partition schemes are 72.46%, 71.38, 70.62, and 71.58, respectively. The sensitivity, precision, specificity, and *F*1-score of the KNN classifier 10-fold partition schemes are 78.62%, 79.34%, 78.62%, and 79.28%, respectively. The sensitivity, precision, specificity, and *F*1-score of the GP classifier 10-fold partition schemes are 90.22%, 89.62%, 88.72%, and 89.48%, respectively. The sensitivity, precision, specificity, and *F*1-score of the RF classifier 10-fold partition schemes are 96.29%, 95.45%, 94.48%, and 95.56%, respectively.  Table 2 represents the performance comparison of our implemented classifiers with other state-of-the-art classifiers.

## 5. Conclusions

The WDBC dataset, which was obtained from the UCI repository, and the classification algorithms such as MLP, KNN, GP, and RF were used in this study. The random forest classifier had the greatest accuracy of 96.24% for breast cancer classification among the four classifiers. Therefore, we conclude that the recommended technique results in classifying probable breast cancer based on the findings.

The limitation of this study is that machine learning is applied to the numeric dataset only. In the future, we try to work on images directly to apply various feature extraction techniques. In addition, we will also try to use deep learning algorithms on the dataset and try to get better classification results.

## Figures and Tables

**Table 1:** Comparison of sensitivity, precision, specificity, and *F*1-score of MLP, KNN, GP, and RF classifiers.

Classifier	Partition scheme	Sensitivity (%)	Precision (%)	Specificity (%)	*F*1 score (%)

MLP	50–50	66.73	65.42	64.58	65.36
	60–40	68.51	67.35	66.34	67.48
	70–30	70.36	69.63	68.21	69.52
	10-fold	72.46	71.38	70.62	71.58

KNN	50–50	74.63	73.26	72.37	73.34
	60–40	76.39	75.57	74.18	75.68
	70–30	78.82	77.26	76.43	77.34
	10-fold	78.62	79.34	78.62	79.28

GP	50–50	84.73	83.62	82.15	83.46
	60–40	86.93	85.76	84.83	85.62
	70–30	88.46	87.25	86.53	87.44
	10-fold	90.22	89.62	88.72	89.48

RF	50–50	90.86	89.49	88.51	89.36
	60–40	92.63	91.38	90.68	91.52
	70–30	94.27	93.67	92.64	93.74
	10-fold	96.29	95.45	94.48	95.56

**Table 2:** Performance comparison with other works from the literature.

Author	Year	Classifier	Accuracy (%)

Quinlan [23]	1996	C4.5	94.74
Hamilton et al. [24]	1996	RAIC	95
Nauck and Kruse [25]	1999	Neuro-fuzzy	95.06
Abonyi and Szeifert [26]	2003	Supervised fuzzy clustering	95.57
Lavanya and Rani [27]	2011	Decision tree algorithms	92.97
Xue et al. [28]	2014	Particle swarm optimization	94.74
**Our study**	**2022**	**Random forest**	**96.24**

## Data Availability

The data are available on request from the corresponding author.
